# Reliability and Validity of Common Subjective Instruments in Assessing Physical Activity and Sedentary Behaviour in Chinese College Students

**DOI:** 10.3390/ijerph19148379

**Published:** 2022-07-08

**Authors:** Hui Gao, Xingxing Li, Yunhua Zi, Xuanwen Mu, Mingjian Fu, Tingting Mo, Kuai Yu

**Affiliations:** Department of Occupational and Environmental Health, Key Laboratory of Environment and Health, Ministry of Education and State Key Laboratory of Environmental Health (Incubating), School of Public Health, Tongji Medical College, Huazhong University of Science and Technology, Wuhan 430030, China; d202081556@hust.edu.cn (H.G.); m201975391@hust.edu.cn (X.L.); m202075478@hust.edu.cn (Y.Z.); d201981374@hust.edu.cn (X.M.); m202175474@hust.edu.cn (M.F.); d201881293@hust.edu.cn (T.M.)

**Keywords:** reliability, validity, accelerometer, physical activity, sedentary behaviour

## Abstract

The reliability and validity of common physical activity (PA) questionnaires are not well investigated in college students. This study aims to evaluate the reliability and validity of common subjective instruments in measuring PA and sedentary behaviour (SB) among college students. A total of 142 college students were included through convenience sampling. Each participant was asked to wear Actigraph wGT3X-BT accelerometers and fill physical activity logs (PAL) for 7 consecutive days. The Global Physical Activity Questionnaire (GPAQ), the International Physical Activity Questionnaire long-form (IPAQ-LF), and short-form (IPAQ-SF) were interviewed by face-to-face at both day 0 and day 8. Reliability was evaluated by intraclass correlation coefficient (ICC), while the validity was evaluated by Spearman correlation coefficient and Bland–Altman statistics. The instruments showed moderate reliability in reporting total PA (ICC = 0.50–0.62) and SB (ICC = 0.47–0.52), while moderate validity in reporting moderate and vigorous PA (MVPA) (*r =* 0.37–0.42), but fair to poor validity in reporting SB (*r =* 0.09–0.28). Bland–Altman plots showed that all the instruments would underestimate MVPA and overestimate SB. Thus, in Chinese younger adults, the GPAQ, IPAQ-LF, IPAQ-SF, and PAL provide limited but acceptable reliability and validity in measuring MVPA and SB, among which GPAQ might be the most valid instrument.

## 1. Introduction

Physical inactivity is a leading risk factor for morbidity and premature mortality worldwide [1,2,3,4]. However, accurate measurement of physical activity (PA) levels has always been a challenge in research aimed to tailor further intervention for at-risk populations [4,5]. Although objective measurement of accelerometer, a motion sensor which has been proved to be comparable to the “gold standard”—doubly labelled water (DLW) method [4,5,6,7,8], has been used in some epidemiological studies recently [9,10,11]. Due to the high cost and time-consuming nature of accelerometers, subjective measurements such as questionnaires and PA logs (PAL) [12], remain the most feasible solution in large-scale population studies [13,14].

During the past decades, several physical activity questionnaires (PAQs) have been developed for PA measurements [15,16]. However, previous validation studies of these questionnaires were mostly conducted in specific occupational populations [17,18,19] (e.g., office workers and nurses), with varied findings. For example, the validity of the Global Physical Activity Questionnaire (GPAQ), a modified version of the International Physical Activity Questionnaire (IPAQ) recommended by the World Health Organization (WHO) for PA surveillance [20], varied from poor to good (*r* = −0.01 to 0.69) for MVPA and (*r* = −0.02 to 0.42) for sedentary behaviour (SB) in comparison with accelerometers [21,22]. Despite the inconsistent validity achieved in previous studies, only two studies have been validated GPAQ among younger adults, who are consistently reported to be at high risk of physical inactivity. One study of 62 college male students in Sandi Arabia showed moderate reliability (ICC = 0.44 to 0.78) and a poor to moderate validity (*r* = 0.08 to 0.32) of GPAQ compared with accelerator [23]. Similarly, the other study of 48 college students of the United Arab Emirates reported moderate reliability (*r* = 0.44 to 0.48) and poor validity (*r* = 0.23) of GPAQ [24]. Despite the small sample size, no studies to date have investigated the reliability and validity of multiple PAQs and PAL simultaneously, especially among younger adults. Furthermore, SB has been reported as a major risk factor for chronic diseases globally [25], there is also a need to develop a valid and reliable instrument that can accurately measure SB levels among younger adults. 

To this end, this study aimed to evaluate the test–retest reliability and validity of PAQs, including GPAQ, IPAQ-LF and IPAQ-SF, and PAL in comparison with wGT3X-BT accelerometer-derived measurements in Chinese college students.

## 2. Methods

### 2.1. Study Participants

A cross-sectional, comparative study was conducted among college students (including undergraduate and graduate students) from Tongji Medical College of Huazhong University of Science and Technology in Wuhan, China from 21 October 2020 to 18 March 2021. Before recruitment, we calculated that the enrolment of 66 participants would achieve adequate statistical power (80%, *α* = 0.05) to detect a moderate correlation (*r* = 0.50) between self-report PA in questionnaires and objective measurements based on accelerometer. In brief, 143 college students (including 57 undergraduate students and 86 graduate students) aged 18–30 years without apparent PA disorders were recruited in the present study through convenience sampling. We further excluded 1 participant who did not participate in the interview of day 8 from the reliability analyses, and 1 participant without more than 5 days of valid accelerometer data (>10 h/day) from the validation analyses. Finally, this study included 142 participants in the reliability analyses and 141 participants in the validity analyses (Supplementary Materials Figure S1). 

### 2.2. Procedures and Variable Definition

The survey lasted for 9 consecutive days. On day 0, trained interviewers measured the demographic characteristics including height and weight of the study participants firstly. Then, they used GPAQ, IPAQ-LF, and IPAQ-SF to obtain information on PA and SB during the past 7 days by face-to-face interviews. After measurements, each participant was instructed to wear an accelerometer on the dominant wrist and to provide a 7-day PAL (Supplementary Materials Figure S2). All of the PAQs, PAL and accelerometer covered the next consecutive 7 days over a 24 h period, initiated on day 1 and finished on day 7. On day 8, all participants were invited to participate in the second interview, during which the above PAQs would be re-administered. Body mass index (BMI; calculated as weight in kilograms divided by height in meters squared) was calculated using measured weight and height.

### 2.3. PAQs

GPAQ [16], IPAQ-LF [15], and IPAQ-SF [15] are used in the present study to collect information on physical activities performed during the last 7 days. Both IPAQ-LF and GPAQ collect the duration and intensity of PA from three domains, including work, transport and leisure, and sedentary time; IPAQ-LF additionally collects information on housework PA. The IPAQ-SF only collects the duration and intensity of PA and sedentary time without distinguishing PA domains (Supplementary Materials Table S1).

### 2.4. PAL

PAL was adapted for this population from existing instruments according to previous studies [26], which was designed to capture a diversity of activities practiced in common daily lives in the term of a 24 h system. The PAL should be filled during the specific consecutive 7 days. Each day, 24 h from 00:00 to 24:00, was separated into 48 half-hour blocks. A total of 10 typical PA types were listed which had been collected in pre-survey of 8 college students (Supplementary Materials Table S2), including sleep, lying down quietly, self-care, sitting and study, walking to work or class, walking as usual, walking at a brisk speed, or backpacking, running with low speed (approximately <160 m/min), running with high speed (approximately ≥160 m/min) [27], eating, and standing quietly. Besides the 10 typical PA types, participants were asked to fill in their custom PA types that performed at least a consecutive 10 min with exact PA type and description: the level of breathing and sweating. For example, playing basketball, taking hard physical effort, or bicycling, making breathe somewhat harder than normal. On day 8, trained interviewers retrieved the 7 day PAL and checked each block for clarity and accuracy. We calculated total vigorous PA (VPA) by summing the PA with energy expenditure (metabolic equivalent tasks [MET]-hour/week) higher than 6 METs according to 2011 compendium of PA, and total moderate PA (MPA) with energy expenditure (MET-hour/week) higher than 3 METs and less than 6 METs (Supplementary Materials Table S3). Finally, we calculated total MVPA by summing the calculated VPA and MPA. We also summarized the total SB (min/week) according to the detailed activities with SB in PAL. 

### 2.5. Accelerometer

Tri-axial ActiGraph wGT3X-BT accelerometers (ActiGraph LLC, Pensacola, FL, USA) were used to assess PA, SB with standard device initialization (sample rate of 60 Hz, 10 s epochs, and normal filter option). In this study, every participant was asked to wear the accelerometer on the dominant wrist for 7 consecutive days except during water activities (e.g., swimming and showering). On day 8 of the survey, the staff retrieved the accelerometers and downloaded the data using the latest version of ActiLife software (version 6.0; ActiGraph, Pensacola, FL, USA). Freedson’s Adult VM3 (2011) [28] cut-off points were used to determine PA at different intensities: light: 0–2690 counts per minute (CPM); moderate: 2691–6166 CPM; vigorous: 6167–9642 CPM; and very vigorous: 9643+ CPM; sedentary behaviour < 100 CPM. Non-wear periods were identified as 60 consecutive minutes with no movement data (0 counts). 

### 2.6. Statistical Analysis

Descriptive characteristics were summarized in terms of means ± standard deviation or percentages (%). The Student’s independent *t*-test and Wilcoxon rank test were used to compare differences for continuous variables, depending on whether the variable was normally distributed, and the Chi-squared test was used for categorical variables. The intraclass correlation coefficients (ICCs) were used to evaluate the test–retest reliability of total PA, domain-specific PA, and intensity-specific PA, as well as SB obtained at day 0 and day 8, and were classified as poor (0.0 to 0.2), fair (0.21 to 0.40), moderate (0.41 to 0.60), strong (0.61 to 0.80) and almost perfect (>0.80) [29]. The Pearson correlation coefficients or Spearman correlation coefficients were used to evaluate the agreement between subjectively (PAQs and PAL) and objectively (accelerometer) measured PA and SB, depending on whether the analysed variable was normally distributed. The correlations were classified as poor, fair, moderate, strong and almost perfect according to the ratings suggested by Landis and Koch [29]. Besides, we used the Bland–Altman plot with 95% limits of agreement to assess the agreement and bias of subjectively (PAQs and PAL) and objectively (accelerometer) measured PA and SB. For sensitivity analysis, we further excluded 28 participants (21.2%) who took off the accelerometer during sleep in a 7-day experiment period to minimize the possibility of poor compliance. All data analyses were conducted using SAS 9.4 and R 3.6.2. A two-sided *p* < 0.05 was considered statistically significant.

All study participants provided written informed consent before the study. The study followed the ethical principles of the Declaration of Helsinki and was approved by the Ethics Committee of Tongji Medical College, Huazhong University of Science and Technology (S033).

## 3. Results

### 3.1. Characteristics of the Participants

The characteristics of 142 participants included in the final analysis are shown in Table 1. Overall, the average age of the participants was 23.7 ± 2.5 years, and 40 (28.2%) were men, 40.1% reported undergraduate degrees. In comparison with women, men were more likely to be graduate students, and have larger BMI than women (*p* < 0.05).

### 3.2. Descriptive Statistics for the PAQs, PAL, and Accelerometer

The descriptive statistics for PAQs, including GPAQ, IPAQ-LF, and IPAQ-SF, PAL, and accelerometer are presented in Table 2. Energy expenditure per week was essentially related to leisure-time PA as well as transport PA and, to a lesser extent, to work. The minutes per week spent on MVPA obtained by PAQs and PAL was two to six times lower than by accelerometer. Specifically, VPA was overestimated, while MPA was underestimated with self-reported PAQs and PAL compared with the accelerometer. As for SB, PAQs and PAL showed two to three times higher duration than accelerometer. The sex-specific descriptive statistics for PAQs, PAL, and accelerometer are presented in Supplementary Materials (Supplementary Materials Table S4). For men, the total PA energy expenditure per week was highly related to leisure-time PA and, to a lesser extent, to transport and work. The time spent on MVPA obtained by PAQs and PAL was lower than those measured by accelerometer, while SB obtained by PAQs and PAL was higher than that measured by accelerometer. As for women, the total PA was related to transport, work, and leisure-time PA. MVPA obtained by PAQs and PAL was lower than those of accelerometer, while SB obtained by PAQs and PAL was higher than that of the accelerometer.

### 3.3. Test–Retest Reliability

As shown in Table 3, GPAQ, IPAQ-LF and IPAQ-SF all showed a moderate test–retest reliability, ranging from 0.50 to 0.62. The ICCs of domain-specific PA ranged from 0.08 to 0.86 for GPAQ and IPAQ-LF, with the highest ICCs observed for leisure PA in GPAQ (0.86). The ICCs of intensity-specific PA ranged from 0.23 to 0.70, with the highest ICCs observed for VPA in IPAQ-SF (0.70). Moderate ICCs of PAQs were also seen for SB. When further exploring the sex-specific reliability, higher ICCs were observed among men than women (Supplementary Materials Table S5).

### 3.4. Concurrent Validity

As presented in Table 4, fair to moderate correlations of MVPA were observed between PAQs, PAL, and accelerometer (*r* = 0.37 to 0.42; all *p* < 0.001). When restricted to intensity-specific validity, similar results were observed for MPV (*r* = 0.35 to 0.40; all *p* < 0.001); however, poor validity was identified for VPA (all *p* > 0.05). We observed poor to fair correlations between PAQs, PAL, and accelerometer for total SB, with the correlation coefficients ranging from 0.15 to 0.28, among which GPAQ showed the highest correlations (*r* = 0.28, 95%CI: 0.12 to 0.42). When further exploring the sex-specific validity, higher correlations were seen among women than men. As for PAL, higher correlations were seen among men in MVPA (0.42 versus 0.38), and similar magnitudes of correlation were seen for SB (−0.45 for men and 0.21 for women; Supplementary Materials Table S6). 

**Figure 1:**
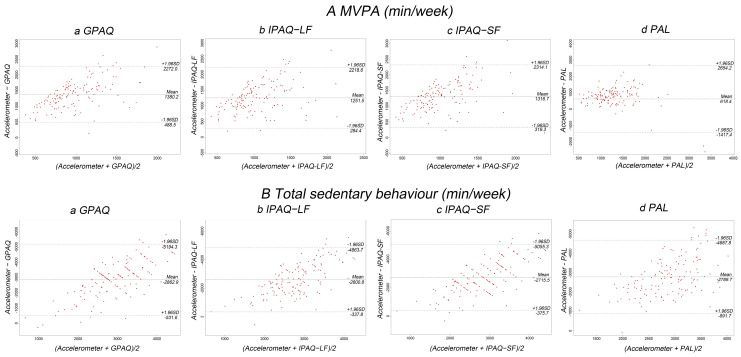
Bland–Altman plots of the validity of the GPAQ, IPAQ-LF, IPAQ-SF, physical activity log and accelerometer. Abbreviation: GPAQ, Global Physical Activity Questionnaire; IPAQ-LF, International Physical Activity Questionn aire-Long Form; IPAQ-SF, International Physical Activity Questionnaire-Short Form; (**A**): Agreement of moderate and vigorous physical activity of questionnaires with accelerometer, for a, GPAQ, b, IPAQ-LF, c, IAPAQ-SF and d, PAL, respectively; (**B**): Agreement of total SB with accelerometer, for a, GPAQ, b, IPAQ-LF, c, IAPAQ-SF and d, PAL, respectively.

The Bland–Altman plot demonstrated the agreement between PAQs, PAL, and accelerometer (Figure 1). Generally, PAQs and PAL underreported the total number of minutes spent on MVPA, with mean differences between GPAQ, IPAQ-LF, IPAQ-SF, and PAL, and accelerometer data of 1380.2 min/week, 1251.5 min/week, 1316.7 min/week, and 618.4 min/week, respectively (Figure 1A). Total SB was overestimated by 2862.9 min/week, 2600.8 min/week, 2715.5 min/week and 2789.7 min/week, respectively (Figure 1B). 

### 3.5. Sensitivity Analysis

Excluding the participants who took off the accelerometer during sleep in the 7-day experiment, the period showed consistent results with our main findings in both reliability and validity analysis. GPAQ showed acceptable reliability, higher reproducibility, for transport PA (ICC = 0.55) than the main results (Supplementary Materials Tables S7 and S8).

## 4. Discussion

This study for the first time evaluated the reliability and validity of PAQs including GPAQ, IPAQ-LF, IPAQ-SF, and PAL simultaneously. All these instruments provided limited and acceptable reliability and validity, among which GPAQ might be the most valid subjective instrument in assessing MVPA and SB in Chinese younger adults.

We found fair to moderate test–retest reliability of three PAQs in reporting total PA and SB, with the highest values obtained through IPAQ-LF, which might be because of the most detailed and concrete nature of IPAQ-LF. The results of IPAQ-SF and GPAQ were generally comparable to previous studies conducted in college students [23,24,30,31]. For example, one study of 133 university students yielded an ICC of 0.52 through repeated IPAQ-SF [30]. Another study of 93 Emirati university students reported the Spearman rho of 0.44 for total SB and 0.78 for MVPA through repeated GPAQ [24], which indicated a good to substantial reliability of GPAQ, the same as this study. Specifically, when concerning different types of PA, the present study showed the best reliability for leisure-time PA and vigorous PA, suggesting the stability for measuring these types of PA. 

Our study showed that PAQs and PAL could achieve a moderate validity for MVPA compared with the accelerometer. We used wGT3x-BT accelerometer as reference to evaluate the validity of PAQs and PAL rather than DLW method, the gold standard for measuring energy expenditure. The DLW method is with high-cost and requires special equipment; besides, it determines only total energy expenditure instead of PA level, let alone frequencies and duration of PA [4]. Furthermore, Chomistek AK et al. and Calabró MA et al. had compared the PA level measured by wGT3x-BT accelerometer with DLW-determined PA and found excellent correlations, indicating the high validity of accelerometer-measured PA [6,7]. According to Bland–Altman plots, the PAQs seemed to underestimate the total MVPA level compared with the accelerometer, which was largely in line with previous reports [21,31]. The underestimated MVPA by PAQs could be explained by the fact that PAQs recorded PA with the duration of at least 10 min, whereas the accelerometer recorded all activities regardless of duration [32]. Notably, the VPA captured by the accelerometer was markedly lower than PAQs in our study, which might be attributed to the discrepancy of intensity between the perception of participants and the criterion standard of PAQs. Individuals might classify their activity as VPA, but actually, the activity they performed did not reach the expected intensity, thus resulting in an overreported VPA duration in PAQs [33]. Among the PAQs examined in this study, GPAQ showed the highest concurrent validity for MVPA, which might be an appropriate choice for future studies aiming to measure PA among young adults. 

Our study also found a fair to poor correlation between PAQs, PAL, and accelerometer for SB. These findings were similar with Cedric Busschaert [34] et al. Their study of 60 Belgian adolescents and 33 adults found that compared with movement monitor (activPAL™), a last 7-day SB questionnaire had a Spearman’s rho of 0.29 in average days (including weekday [*r* = 0.42] and weekdays [*r* = 0.002]) and 0.49 (weekday: *r* = 0.52 and weekend: *r* = 0.06), respectively. Additionally, our study found that the PAQs and PAL significantly overestimated total SB in comparison with that measured by accelerometer, which was in line with the study conducted by Michelle Laeremans et al. [35]. This study reported that GPAQ had poor to fair validity for SB (*r* = 0.09 to 0.25) compared with SenseWear accelerometer (model MF-SW, Body Media, Pittsburgh, PA, USA). Besides, Maria Hagstromer et al. [36] also reported that compared with accelerometer, IPAQ-LF had a fair correlation for sitting (*r* = 0.23). The overestimated SB by PAQs may be explained by the double-reporting resulting from the simultaneous behaviours, for example sitting while watching TV [34]. However, future studies are still needed to validate these results.

By strictly adherence to standardized WHO protocols, this is the first study, to our knowledge, to validate the reliability and validity of the three most common PAQs, including IPAQ-LF, IPAQ-SF, and GPAQ, and PAL simultaneously among free-living Chinese college students. This study also has several limitations. First, the present study used accelerometer as a validity criterion rather than the gold standard such as DLW; however, DLW is seldomly used in population studies because of its high cost and inconvenience nature. Second, study participants were recruited by convenient sampling from medical colleges, representative samples with varying PA levels should be used in future studies. Third, the consecutively 7-day PAL might strengthen the memory, making it relatively easier when conducting the day 8 questionnaires. However, this could reduce the recall bias of our test to some extent and validate our results. Finally, despite being carefully controlled, the Hawthorne effect [37] and recall bias [38] could not be eliminated.

## 5. Conclusions

In Chinese college students, both PAQs and PAL could provide limited but acceptable reliability and validity for the measurement of PA, especially for MVPA, and the total SB might be overreported by PAQs and PAL. Overall, GPAQ, with the strengths of convenience, time-effective, and moderate validity, might be a recommended instrument for measuring MVPA and SB among college students. 

## Figures and Tables

**Table 1:** Baseline characteristics of study participants (*n* = 142).

Characteristics *	Total	Men	Women	*p* ^a^
N	N = 142	N = 40	N = 102	
Age (years)	23.7 ± 2.5	24.1 ± 3.6	23.6 ± 2.0	0.96
Education, n (%)				0.04
Undergraduate	57 (40.1)	20 (50.0)	37 (36.3)	
Graduate	85 (59.9)	20 (50.0)	65 (67.7)	
BMI (kg/m^2^)	21.8 ± 3.0	23.5 ± 2.9	21.1 ± 2.8	<0.0001
BMI classes (kg/m^2^)				<0.0001
Underweight < 18.5	17 (12.0)	1 (2.5)	16 (15.7)	
Acceptable 18.5–23.9	98 (69.0)	22 (55.0)	76 (74.5)	
Overweight > 23.9	27 (19.0)	17 (42.5)	10 (9.8)	

* Number (percentage) and mean (standard deviation) are presented for categorical and continuous variables, respectively. ^a^ *p* values are obtained from student’s *t* test for normal distribution variables and nonparametric test for skewed distribution.

**Table 2:** Data for physical activity and sedentary behaviour measured by GPAQ, IPAQ-LF, IPAQ-SF, PAL and accelerometer.

	GPAQ		IPAQ-LF		IPAQ-SF		PAL	Accelerometer
	D0	D8	D0	D8	D0	D8		
	Mean ± SD *	Mean ± SD *	Mean ± SD *	Mean ± SD *	Mean ± SD *	Mean ± SD *	Mean ± SD *	Mean ± SD *
**Total PA** **(MET min/week)**	1632.2 ± 1873.5	1439.2 ± 1421.6	1887.3 ± 1671.2	1871.3 ± 1664.9	1683.5 ± 1770.6	1520.0 ± 1410.0	n/a	n/a
**MVPA by domain (MET min/week)**								
Work	360.3 ± 1191.7	279.6 ± 871.7	288.5 ± 721.9	314.3 ± 731.0	n/a	n/a	n/a	n/a
Transport	666.4 ± 862.2	575.7 ± 557.6	698.9 ± 620.4	636.0 ± 661.9	n/a	n/a	n/a	n/a
Household	n/a	n/a	69.9 ± 132.3	113.2 ± 363.3	n/a	n/a	n/a	n/a
Leisure	598.2 ± 1302.7	580.8 ± 1023.5	830.0 ± 1310.0	807.9 ± 1096.0	n/a	n/a	n/a	n/a
**MVPA by intensity (min/week)**								
MVPA	360.6 ± 422.7	309.1 ± 290.6	417.5 ± 337.8	434.8 ± 372.7	402.2 ± 406.3	370.0 ± 334.4	1069.1 ± 985.7	1687.5 ± 489.0
MPA	295.7 ± 395.7	258.3 ± 264.6	353.6 ± 303.2	377.5 ± 351.2	335.0 ± 387.8	315.3 ± 321.1	892.8 ± 631.4	1686.3 ± 490.3
VPA	65.0 ± 169.9	50.4 ± 116.7	63.9 ± 154.8	57.3 ± 126.1	67.2 ± 164.8	54.8 ± 123.0	176.3 ± 408.4	1.0 ± 12.8
**Total SB (min/week)**	4181.5 ± 1195.5	4067.5 ± 1140.8	4237.7 ± 1258.1	4327.9 ± 1213.5	3873.7 ± 1158.7	4181.5 ± 1195.5	4264.5 ± 877.5	1474.8 ± 500.8

Abbreviations: GPAQ, Global Physical Activity Questionnaire; IPAQ-LF, International Physical Activity Questionnaire-Long Form; IPAQ-SF, International Physical Activity Questionnaire-Short Form; MET, metabolic equivalent task; PA, physical activity; SB, sedentary behaviour; MVPA, moderate and vigorous physical activity; n/a, not accessed by the questionnaire; n/a, not accessed by the instrument. * Continuous variables were presented as mean ± SD.

**Table 3:** Test–retest reliability of GPAQ, IPAQ-LF and IPAQ-SF (*n* = 141).

	GPAQ	IPAQ-LF	IPAQ-SF
Variables	ICC (95%CI)	ICC (95%CI)	ICC (95%CI)
**Total PA (MET min/week)**	0.50 (0.38, 0.62)	0.62 (0.52, 0.72)	0.55 (0.44, 0.66)
**MVPA by domain (MET min/week)**			
Work	0.28 (0.16, 0.45)	0.25 (0.13, 0.43)	n/a
Transport	0.32 (0.20, 0.48)	0.21 (0.09, 0.41)	n/a
Household	n/a	0.08 (0.01, 0.45)	n/a
Leisure	0.86 (0.81, 0.90)	0.66 (0.56, 0.74)	n/a
**MVPA by intensity (min/week)**			
MVPA	0.31 (0.18, 0.47)	0.53 (0.42, 0.65)	0.37 (0.24, 0.52)
MPA	0.23 (0.11, 0.42)	0.46 (0.33, 0.59)	0.27 (0.14, 0.44)
VPA	0.60 (0.49, 0.70)	0.69 (0.60, 0.77)	0.70 (0.61, 0.78)
**Total SB (min/week)**	0.47 (0.35, 0.60)	0.54 (0.43, 0.66)	0.52 (0.40, 0.63)

Abbreviations: GPAQ, Global Physical Activity Questionnaire; IPAQ-LF, International Physical Activity Questionnaire-Long Form; IPAQ-SF, International Physical Activity Questionnaire-Short Form; MET, metabolic equivalent task; PA, physical activity; SB, sedentary behaviour; MVPA, moderate and vigorous physical activity; ICC, intraclass correlation coefficient; CI, confidence interval; n/a, not accessed by this instrument.

**Table 4:** Concurrent validity data of the GPAQ, IPAQ-LF, IPAQ-SF and PAL (*n* = 141).

	Spearman’s Rho (95%CI) Compared with Accelerometer			
	GPAQ	IPAQ-LF	IPAQ-SF	PAL
**MVPA**	0.42 (0.27–0.55)	0.37 (0.22–0.50)	0.37 (0.22–0.50)	0.40 (0.25–0.53)
MPA	0.40 (0.25–0.53)	0.35 (0.19–0.48)	0.34 (0.19–0.48)	0.40 (0.25–0.53)
VPA *	−0.06 (−0.22–0.11)	−0.07 (−0.23–0.10)	−0.06 (−0.22–0.11)	0.40 (−0.13–0.20)
**Total SB**	0.28 (0.12–0.42)	0.21 (0.04–0.37)	0.17 (0.003–0.32)	0.09 (−0.08–0.25)

Abbreviations: CI, confidence interval; GPAQ, Global Physical Activity Questionnaire; IPAQ-LF, International Physical Activity Questionnaire-Long Form; IPAQ-SF, International Physical Activity Questionnaire-Short Form; PAL, physical activity log; CI, confidence interval; PA, physical activity; SB, sedentary behaviour; MVPA, moderate and vigorous physical activity. * Less than 10% of the participants with VPA captured by the accelerometer.

## Data Availability

The dataset from the current analysis is not public, but is available from the corresponding author upon reasonable request.

## Supplementary Materials

The following supporting information can be downloaded at: https://www.mdpi.com/article/10.3390/ijerph19148379/s1, Figure S1: Flowchart of the study. Figure S2: The physical activity log in the study. Table S1: The physical activity types collected by physical activity log in the study. Table S2: The typical physical activities shown in pre-survey. Table S3: The METs for specific physical activity in GPAQ, IPAQ-LF and IPAQ-SF. Table S4: Sex-specific data for physical activity measured by GPAQ, IPAQ-LF and IPAQ-SF. Table S5: Sex-specific test–retest reliability of GPAQ, IPAQ-LF and IPAQ-SF. Table S6: Sex-specific concurrent validity data of the GPAQ, IPAQ-LF, IPAQ-SF and PAL. Table S7: Sensitivity analysis for test–retest reliability of GPAQ, IPAQ-LF and IPAQ-SF (n = 115). Table S8: Sensitivity analysis for concurrent validity data of the GPAQ, IPAQ-LF, IPAQ-SF and PAL (n = 115).

## Abbreviations

PA, physical activity; SB, sedentary behaviour; PAL, physical activity logs; GPAQ, Global Physical Activity Questionnaire; IPAQ-LF, International Physical Activity Questionnaire long-form; IPAQ-SF, International Physical Activity Questionnaire short-form; ICC, intraclass correlation coefficient; MVPA, moderate and vigorous physical activity; PAQ, physical activity questionnaire; DLW, doubly labeled water; WHO, World Health Organization; VPA, vigorous physical activity; MET, metabolic equivalent task; MPA, moderate physical activity.
