# Supplementary material for: Reliability and Validity of Common Subjective Instruments in Assessing Physical Activity and Sedentary Behaviour in Chinese College Students
Source: Int J Environ Res Public Health. 2022 Jul 8;19(14):8379. doi: 10.3390/ijerph19148379 (PMC9320576; doi:10.3390/ijerph19148379)
Supplement: Supplementary file 1 [file ijerph-19-08379-s001.zip › ijerph-1752826-supplementary.pdf]

Supplementary Materials

Figure S1. Flowchart of the Study.
Figure S2. The Physical Activity Log in the Study.
Table S1. The Physical Activity Types Collected by Physical Activity Log in the Study.
Table S2. The Typical Physical Activities Showed in Pre-survey.
Table S3. The METs for Specific Physical Activity in GPAQ, IPAQ-LF and IPAQ-SF.
Table S4. Sex-specific Data for PA Measured by GPAQ, IPAQ-LF and IPAQ-SF.
Table S5. Sex-specific Test-retest Reliability of GPAQ, IPAQ-LF and IPAQ-SF.
Table S6. Sex-specific Concurrent Validity Data of the GPAQ, IPAQ-LF, IPAQ-SF and PAL.
Table S7. Sensitivity Analysis for Test-retest Reliability of GPAQ, IPAQ-LF and IPAQ-SF (n=115).
Table S8. Sensitivity Analysis for Concurrent Validity Data of the GPAQ, IPAQ-LF, IPAQ-SF and PAL (n=115).

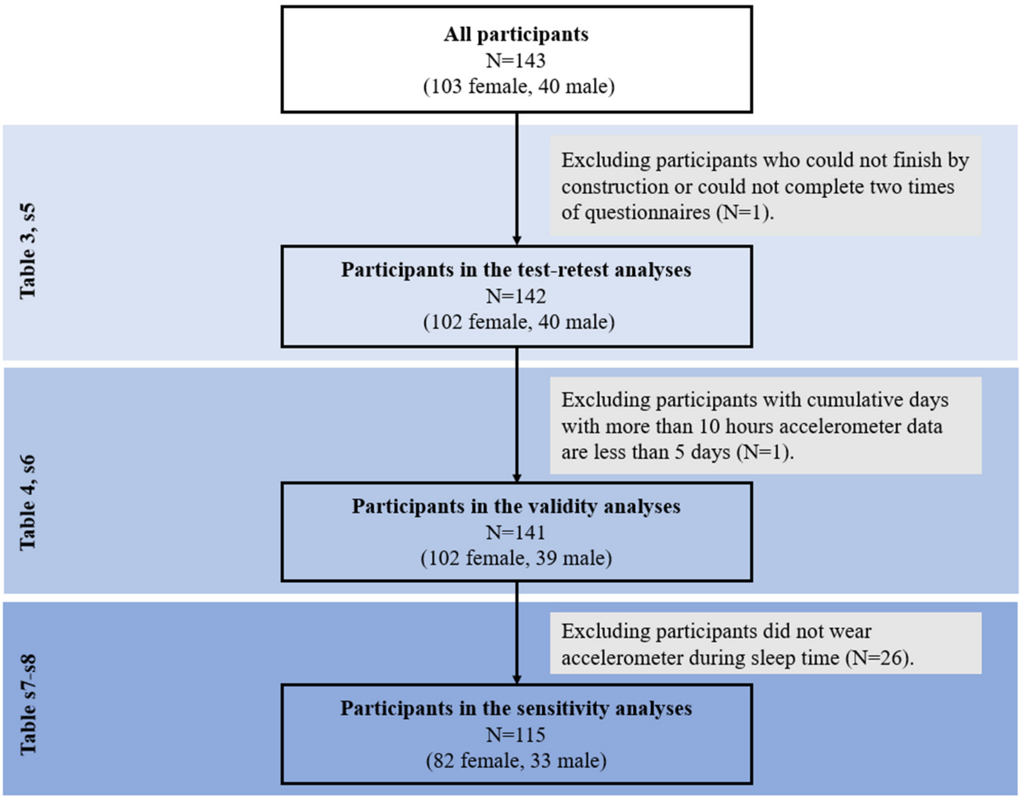

Figure S1. Flowchart of the Study

The information only served for research and refused any other purpose.

Physical activity log

Name ________ Number of waring day _______ Date_________

First, record any physical activity that lasted for more than 10 mins, and fill in the blanks.
Tips: 1, For your convenience, fill in the corresponding **code** of the following physical activity.
2, We listed 10 typical physical activity types, and please added your tailored physical activities with **types and subjective feeling** when doing other physical activities.

For example,

0, sleep **3**, sedentary behavior **5**, walk to class

07:00-07:30	**0**	07:30-08:00	**5**
08:00-08:30	Yoga, sweat	08:30-09:00	**5**(10) +**3**(20)

Explanation:
7:00-7:30 slept;
7:30-8:00 walked to class.
8:00-8:30 yoga with sweet;
8:30-9:00 walked (ten mins) and then sat (20 mins.)

Physical activity code
0 sleep
1 Lying down quietly
2 Self care
3 Sitting and study
4 Walk to work or class
5 Walk as usual
6 Walk, brisk speed or backpacking
7 Running, 3mins/400M
8 Running, 2mins/400M
9 Eating
10 Standing, quietly

Please, refer to the left example to fill in.

00:00-00:30		00:30-01:00	
01:00-01:30		01:30-02:00	
02:00-04:00		04:00-06:00	
06:00-06:30		06:30-07:00	
07:00-07:30		07:30-08:00	
08:00-08:30		08:30-09:00	
09:00-09:30		09:30-10:00	
10:00-10:30		10:30-11:00	
11:00-11:30		11:30-12:00	
12:00-12:30		12:30-13:00	
13:00-13:30		13:30-14:00	
14:00-14:30		14:30-15:00	
15:00-15:30		15:30-16:00	
16:00-16:30		16:30-17:00	
17:00-17:30		17:30-18:00	
18:00-18:30		18:30-19:00	
19:00-19:30		19:30-20:00	
20:00-20:30		20:30-21:00	
21:00-21:30		21:30-22:00	
22:00-22:30		22:30-23:00	
23:00-23:30		23:30-24:00	
00:00-00:30		00:30-01:00	

Second, fill in the **time in bed** and **time out of bed** of this day (try not conclude the lying time).

Time in bed		Time out of bed	

Third, whether take off the accelerometer during this day? ☐Yes ☐No.
If yes, please fill in the **Take off time** and **Back on time**.

Take off time		Back on time	
Take off time		Back on time	

Figure S2. The Physical Activity Log in the Study

Table S1. The METs for Specific Physical Activity in GPAQ, IPAQ-LF and IPAQ-SF.

Characteristics	PA feature (intensity, type, etc.)	MET[#]s
**GPAQ**		
Work	vigorous	8
	moderate	4
Transport	moderate	4
Leisure	vigorous	8
	moderate	4
Sedentary	do not related to light intensity	1
**IPAQ-LF**		
Work	walking	3.3
	moderate	4
	vigorous	8
Transport	by car or bus	1.3
	walking	3.3
	cycling	4
Housework	moderate-indoor	3.5
	moderate-outdoor	4
	vigorous-outdoor	6
Leisure	walking	3.3
	moderate	4
	vigorous	8
Sedentary	do not related to light intensity	1
**IPAQ-SF**		
Vigorous	carrying heavy loads, swimming, etc.	8
Moderate	cycling, playing table tennis, etc.	4
Walking	when working, home, transport, etc.	3.3
Sedentary	when working, home, transport	1

Notes: Abbreviation: METs: metabolic equivalent tasks; PA, physical activity; GPAQ, Global Physical Activity Questionnaire; IPAQ-LF, International Physical Activity Questionnaire-Long Form; IPAQ-SF, International Physical Activity Questionnaire-Short Form.

[#], The METs were determined according to 2011 compendium of physical activities: A second update of codes and MET values.

Table S2. The Typical Physical Activities Showed in Pre-survey.

Activity	Frequency (Day/week)
sleep	7.0
lying down quietly	7.0
Self-care	7.0
sedentary and study	7.0
walking	7.0
eating	7.0
standing, quiet	4.4
walk to work or class	3.9
walk, brisk speed or backpacking	1.1
cleaning	0.7
washing clothes	0.6
dancing	0.4
cycling	0.3
playing table tennis	0.3
running, 3mins/400M	0.1
running, 2mins/400M	0.0

Notes: A total of 7 younger adults finished the pre-survey filling consecutive 7-day PAL. We choose the PA type with frequency more than 1 day/week as typical PA type of younger adults. Besides, we choose running with different speed as example to deepen the understanding of intensity of custom PA type for participants.

Table S3. The Physical Activity Types Collected by Physical Activity Log in the Study.

Physical Activity Types	Code in 2011 Compendium	METs
The Typical Physical Activity Types.		
sleep	7030	1
lying down quietly	7010	1.3
self-care	13040	2
sedentary and study	9060	1.3
walk to work or class	17270	4
walking	17170	3
walk, brisk speed or backpacking#	11793, 17010	5.25
running, 3mins/400M	12030	8.3
running, 2mins/400M	12080	11.5
eating	13030	1.5
standing, quiet	1303	1.5
The Custom Physical Activity		
showering	13050	2
bicycling for leisure and work	1010	4
cleaning, straightening up, general light effort	5040	2.5
fold and hanging, put clothes in washer	5090	2
riding in car, bus, train#	16015, 16016	1.3
eliminating, squating	13009	1.8
sweeping floor, home cleaning, general	5010	3.3
chemistry lab work	11580	1.5
combination of jog/walk	12010	6
dancing	3031	7.8
swimming	15140	4
walking for pleasure	17160	3.5
conditioning exercise, with trainer#	2052, 2048	5
soccer, general	15610	7
hula loop	2045	3.5
basketball, general	15050	6
basketball, officiating	15060	7
video exercise workouts#	2143, 2146	5
singing, sedentary and standing#	20005, 20020	1.9
badminton, general	15030	5.5
stretching	2101	2.3
table tennis	15660	4
high intensity internal training, circuit training	2040	8
washing by hand	5092	4
electric motor bike#	1010, 16016	2.65
miscellaneous, internship, tutor, quiet	9071	2.5
carrying heavy loads, occupational	11050	8
kitchen activity, general	5035	3.3
guitar, sedentary	10120	2
climbing hills with light bag	17035	6.6
tennis, general	15695	5
Chinese lute, violin, sedentary	10100	2
volleyball	15710	4

miscellaneous, talking in person, on a phone	9050	1.8
rope jumping, slow pace	15552	8.8
rollerblading	15591	7.5
Push-ups, sit ups, conditioning exercise	2022	3.8
billiards	15080	2.5
low intensity		2
moderate intensity		4
vigorous intensity		8

Notes: Abbreviation: METs: metabolic equivalent tasks.
[#], The physical activities concerned with two or more physical activity Code in 2011 Compendium, the METs was calculated by the means of physical activity codes.

Table S4. Sex-specific Data for Physical Activity Measured by GPAQ, IPAQ-LF, IPAQ-SF, PAL and Accelerometer.

	GPAQ		IPAQ-LF		IPAQ-SF		PAL	Accelerometer
	D0	D8	D0	D8	D0	D8		
	Mean±SD*	Mean±SD*	Mean±SD*	Mean±SD*	Mean±SD*	Mean±SD*	Mean±SD*	Mean±SD*
Men								
**Total PA (MET min/week)**	1930.0±2138.4	1706.5±1695.8	2386.3±2410.1	2077.2±1959.8	2123.3±2126.2	1722.6±1799.5	n/a	n/a
**MVPA by domain (MET min/week)**								
Work	237.0±624.6	301.5±759.6	320.5±785.4	152.2±473.0	n/a	n/a	n/a	n/a
Transport	453.5±555.2	451.5±444.1	441.2±392.9	578.4±484.5	n/a	n/a	n/a	n/a
Household	n/a	n/a	63.1±123.2	125.4±334.8	n/a	n/a	n/a	n/a
Leisure	1239.5±1979.9	953.5±1439.8	1561.5±2017.9	1221.2±1564.7	n/a	n/a	n/a	n/a
**MVPA duration by intensity (min/week)**								
MVPA	400.2±362.8	327.0±294.1	453.5±387.9	414.1±337.5	390.9±285.1	356.7±352.8	1062.2±1151.6	1517.0±451.5
MPA	221.5±229.8	247.3±225.6	298.0±271.8	293.1±254.9	222.6±157.2	248.0±296.8	832.9±725.0	1517.1±451.5
VPA	178.7±283.7	89.7±169.8	155.6±255.6	121.0±189.2	168.2±267.0	108.6±183.5	229.3±458.6	0.0±0.0
**Total SB (min/week)**	4488.8±1291.6	4450.3±1457.0	4090.5±1125.7	4383.0±1246.3	4163.3±1249.1	4520.3±1294.5	4773.6±975.1	1587.0±520.4
Women								
**Total PA (MET min/week)**	1514.3±1755.3	1333.4±1291.5	1691.2±1232.4	1790.6±1537.3	1511.0±1588.7	1440.6±1225.9	n/a	n/a
**MVPA by domain (MET min/week)**								
Work	408.6±1350.4	271.0±915.3	276.0±699.2	377.8±803.3	n/a	n/a	n/a	n/a
Transport	749.9±945.3	624.4±591.1	800.0±664.1	658.6±720.5	n/a	n/a	n/a	n/a
Household	n/a	n/a	72.6±136.1	108.4±375.4	n/a	n/a	n/a	n/a
Leisure	344.2±785.7	433.2±761.9	543.2±727.0	645.8±798.4	n/a	n/a	n/a	n/a
**MVPA duration by intensity (min/week)**								
MVPA	345.1±444.7	298.0±290.0	403.5±317.0	442.9±387.0	406.7±446.1	375.3±328.5	1071.7±920.7	1752.6±489.1
MPA	324.7±441.8	262.7±279.5	375.5±313.2	410.6±378.4	379.0±439.9	341.6±327.8	915.7±594.1	1751.0±491.1

VPA	20.4±46.0	34.9±83.9	28.0±59.8	32.3±78.3	27.6±69.1	33.7±80.9	156.1±388.0	1.5±15.0
**Total SB (min/week)**	4139.3±1237.2	4279.9±1108.1	3764.4±1188.0	3943.8±1078.0	3760.1±1107.1	4048.6±1133.5	4069.9±755.9	1431.9±489.0

Abbreviations: GPAQ, Global Physical Activity Questionnaire; IPAQ-LF, International Physical Activity Questionnaire-Long Form; IPAQ-SF, International Physical Activity Questionnaire-Short Form; MET, metabolic equivalent task; PA, physical activity; SB, sedentary behaviour; MVPA, moderate and vigorous physical activity; n/a, not accessed by the instrument.

[*] Mean±Standard Deviation is presented for the continues variables in this table.

Table S5. Sex-specific Test-retest Reliability of GPAQ, IPAQ-LF and IPAQ -SFSF.

	Intraclass correlation coefficient (95%CI) for day0 and day8					
	GPAQ		IPAQ-LF		IPAQ-SF	
	Men	Women	Men	Women	Men	Women
**Total PA (MET min/week)**	0.81 (0.68, 0.89)	0.30 (0.15, 0.50)	0.84 (0.73, 0.91)	0.40 (0.26, 0.57)	0.84 (0.72, 0.91)	0.33 (0.18, 0.52)
**MVPA by domain (MET min/week)**						
Work	0.33 (0.12, 0.63)	0.27 (0.13, 0.48)	0.60 (0.40, 0.78)	0.15 (0.04, 0.44)	n/a	n/a
Transport	0.43 (0.21, 0.68)	0.29 (0.15, 0.49)	0.17 (0.02, 0.63)	0.19 (0.07, 0.44)	n/a	n/a
Household	n/a	n/a	0.25 (0.07, 0.61)	0.02 (0.00, 0.99)	n/a	n/a
Leisure	0.89 (0.81, 0.94)	0.78 (0.69, 0.85)	0.80 (0.67, 0.89)	0.24 (0.11, 0.46)	n/a	n/a
**MVPA duration by intensity (min/week)**						
MVPA	0.61 (0.41, 0.78)	0.21 (0.08, 0.45)	0.78 (0.64, 0.88)	0.43 (0.29, 0.59)	0.66 (0.47, 0.81)	0.29 (0.15, 0.49)
MPA	0.27 (0.08, 0.61)	0.21 (0.08, 0.45)	0.64 (0.45, 0.80)	0.41 (0.26, 0.57)	0.15 (0.02, 0.65)	0.26 (0.12, 0.47)
VPA	0.63 (0.43, 0.79)	0.20 (0.08, 0.45)	0.81 (0.69, 0.90)	-*	0.78 (0.63, 0.88)	0.24 (0.10, 0.46)
**Total SB (min/week)**	0.64 (0.44, 0.80)	0.38 (0.23, 0.55)	0.56 (0.35, 0.75)	0.52 (0.39, 0.66)	0.61 (0.40, 0.78)	0.45 (0.30, 0.60)

Abbreviations: GPAQ, Global Physical Activity Questionnaire; IPAQ-LF, International Physical Activity Questionnaire-Long Form; IPAQ-SF, International Physical Activity Questionnaire-Short Form; MET, metabolic equivalent task; PA, physical activity; SB, sedentary behaviour; MVPA, moderate and vigorous physical activity; CI, confidence interval; n/a, not accessed by the instrument.

*, do not accessed due to infrequently engagement.

Table S6. Sex-specific Concurrent Validity of the GPAQ, IPAQ-LF, IPAQ-SF and PAL.

	Spearman's Rho (95%CI) compared with accelerometer			
	**GPAQ**	**IPAQ-LF**	**IPAQ-SF**	**PAL**
**Men**				
MVPA	0.26 (-0.06-0.53)	0.26 (-0.07-0.53)	0.25 (-0.07-0.52)	0.42 (0.12-0.65)
MPA	0.30 (-0.02-0.56)	0.19 (-0.13-0.48)	0.12 (-0.20-0.42)	0.38 (0.08-0.62)
VPA*	-	-	-	-
Total SB	0.09 (-0.23-0.39)	-0.09 (-0.41-0.24)	-0.002 (-0.32, 0.32)	-0.45 (-0.67--0.16)
**Women**				
MVPA	0.49 (0.33-0.63)	0.41 (0.23-0.56)	0.40 (0.23-0.55)	0.38 (0.20-0.54)
MPA	0.43 (0.25-0.58)	0.37 (0.19-0.53)	0.39 (0.21-0.54)	0.38 (0.20-0.53)
VPA*	-0.06 (-0.25-0.14)	-0.06 (-0.25-0.13)	-0.06 (-0.25-0.14)	0.07 (-0.13-0.26)
Total SB	0.36 (0.17-0.52)	0.31 (0.11-0.47)	0.22 (0.03-0.40)	0.21 (0.01-0.38)

Abbreviations: CI, confidence interval; GPAQ, Global Physical Activity Questionnaire; IPAQ-LF, International Physical Activity Questionnaire-Long Form; IPAQ-SF, International Physical Activity Questionnaire-Short Form; PAL, physical activity log; CI, confidence interval; PA, physical activity; SB, sedentary behaviour; MVPA, moderate and vigorous physical activity.

* Less than 10% of the participants with VPA captured by the accelerometer.

Table S7. Sensitivity Analysis for Test-retest Reliability of GPAQ, IPAQ-LF and IPAQ-SF (n=115).

	GPAQ	IPAQ-LF	IPAQ-SF
Variables	ICC (95%CI)	ICC (95%CI)	ICC (95%CI)
**Total PA (MET min/week)**	0.56 (0.43, 0.68)	0.64 (0.52, 0.74)	0.59 (0.46, 0.70)
**MVPA by domain (MET min/week)**			
Work	0.30 (0.16, 0.48)	0.23 (0.10, 0.44)	n/a
Transport	0.55 (0.42, 0.67)	0.27 (0.13, 0.47)	n/a
Household	n/a	0.06 (0.00, 0.60)	n/a
Leisure	0.87 (0.82, 0.91)	0.68 (0.57, 0.77)	n/a
**MVPA duration by intensity (min/week)**			
MVPA	0.35 (0.21, 0.52)	0.54 (0.41, 0.66)	0.40 (0.26, 0.56)
MPA	0.28 (0.14, 0.47)	0.46 (0.32, 0.60)	0.29 (0.15, 0.48)
VPA	0.74 (0.65, 0.81)	0.74 (0.65, 0.81)	0.72 (0.62, 0.80)
**Total SB (min/week)**	0.44 (0.30, 0.59)	0.53 (0.40, 0.66)	0.51 (0.37, 0.64)

Abbreviations: GPAQ, Global Physical Activity Questionnaire; IPAQ-LF, International Physical Activity Questionnaire-Long Form; IPAQ-SF, International Physical Activity Questionnaire-Short Form; MET, metabolic equivalent task; PA, physical activity; SB, sedentary behaviour; MVPA, moderate and vigorous physical activity; ICC, intraclass correlation coefficient; CI, confidence interval, n/a, not accessed by the questionnaire.

Table S8. Sensitivity Analysis for Concurrent Validity Data of the GPAQ, IPAQ-LF, IPAQ-SF and PAL (n=115).

	Spearman's Rho (95%CI) compared with accelerometer			
	**GPAQ**	**IPAQ-LF**	**IPAQ-SF**	**PAL**
**MVPA**	0.40 (0.23-0.54)	0.37 (0.20-0.52)	0.34 (0.17-0.50)	0.34 (0.17-0.50)
**MPA**	0.38 (0.21-0.53)	0.31 (0.14-0.47)	0.32 (0.15-0.48)	0.34 (0.16-0.49)
**VPA***	-0.07 (-0.25-0.11)	-0.08 (-0.25-0.11)	-0.07 (-0.25-0.12)	0.04 (-0.15-0.22)
**Total SB**	0.26 (0.08-0.43)	0.15 (-0.03-0.33)	0.20 (0.02-0.37)	-0.02 (-0.21-0.16)

Abbreviations: CI, confidence interval; GPAQ, Global Physical Activity Questionnaire; IPAQ-LF, International Physical Activity Questionnaire-Long Form; IPAQ-SF, International Physical Activity Questionnaire-Short Form; PAL, physical activity log; PA, physical activity; SB, sedentary behaviour; MVPA, moderate and vigorous physical activity.

* Less than 10% of the participants with VPA captured by the accelerometer.
